# Ensembling U-Nets for microaneurysm segmentation in optical coherence tomography angiography in patients with diabetic retinopathy

**DOI:** 10.1038/s41598-024-72375-2

**Published:** 2024-09-14

**Authors:** Lennart Husvogt, Antonio Yaghy, Alex Camacho, Kenneth Lam, Julia Schottenhamml, Stefan B. Ploner, James G. Fujimoto, Nadia K. Waheed, Andreas Maier

**Affiliations:** 1 Pattern Recognition Lab, Friedrich-Alexander-Universität Erlangen-Nürnberg, 91058 Erlangen , Germany; 2 New England Eye Center, Tufts School of Medicine, Boston, MA 02111 USA; 3 Research Laboratory of Electronics, Massachusetts Institute of Technology, Cambridge, MA 02139 USA

**Keywords:** Machine learning, Eye diseases, Retinal diseases, Eye manifestations

## Abstract

Diabetic retinopathy is one of the leading causes of blindness around the world. This makes early diagnosis and treatment important in preventing vision loss in a large number of patients. Microaneurysms are the key hallmark of the early stage of the disease, non-proliferative diabetic retinopathy, and can be detected using OCT angiography quickly and non-invasively. Screening tools for non-proliferative diabetic retinopathy using OCT angiography thus have the potential to lead to improved outcomes in patients. We compared different configurations of ensembled U-nets to automatically segment microaneurysms from OCT angiography fundus projections. For this purpose, we created a new database to train and evaluate the U-nets, created by two expert graders in two stages of grading. We present the first U-net neural networks using ensembling for the detection of microaneurysms from OCT angiography en face images from the superficial and deep capillary plexuses in patients with non-proliferative diabetic retinopathy trained on a database labeled by two experts with repeats.

## Introduction

Diabetic retinopathy (DR) is one of the leading causes of blindness among working-age individuals worldwide. It consists of two stages, an earlier non-proliferative (NPDR) stage, and a more advanced proliferative stage (PDR) which occurs when new retinal blood vessels form (‘proliferate’) often in response to tissue retinal ischemia. During the earlier NPDR stage, patients may be asymptomatic, however microaneurysms (MAs), the hallmark of this stage, already emerge as outpouchings of the retinal blood vessels that are weakened as a result of the sugar overload in the blood^1–4^.

NPDR can be graded as mild, moderate, and severe and MAs are an early and important clinical sign of disease progression and are a main component of classifying DR severity. Early diagnosis of DR is key for treatment and preserving patient vision since it can prevent blindness in more than 90% of patients^1^.

Fluorescein angiography (FA) is currently the gold standard for the diagnosis of DR and the most sensitive test for detecting MAs. However, it suffers from several drawbacks. During FA imaging, fluorescein, a contrast agent, is injected to highlight the patient’s retinal vasculature^3^. In rare cases, fluorescein can lead to an anaphylactic shock in patients that are allergic to it, a reaction that can be fatal if urgent medical intervention is not taken^2^. This makes FA invasive, costly, and time consuming. Furthermore, superposition of retinal capillary layers and leakage pose a challenge to FA, while the deep capillary plexus is barely visible in FA^2,5^. The combination of these factors makes FA less suitable as an ideal screening tool for DR, pushing scientists and engineers to find complementary imaging modalities such as optical coherence tomography angiography (OCTA)^3,6^. OCTA allows the separation of the superficial (SCP) and deep capillary plexuses (DCP) and does not require injections of a contrast agent. OCTA on the other hand does not show all MAs visible with FA as the speed of blood flow within certain MAs is below the threshold of OCTA detection^2^.

Machine learning and deep learning methods for biomedical segmentation tasks have made significant progress over the last decade. This ranges from segmentation of disease markers on acquired clinical images to patient referral^7,8^. The U-net architecture in particular has been successful in the field of biomedical segmentation tasks and can be considered state-of-the-art^9^. Three-dimensional U-nets have been trained by Deep Mind to enable the referral of patients based on OCT scans^8^.

There already exists an expansive body of work in the scientific literature about finding MAs or markers of DR in images. This includes using non deep learning-related approaches such as eigenvalue analysis, radon-transform, multi-agent learning, and dictionary learning on fundus photos^10–14^. Artificial neural networks have been used to locate MAs in fundus photos^15–20^ as well as in FA fundus images^21,22^. The U-net architecture has also been used to segment MAs in fundus photos^23–26^. Neural networks have also been used in conjunction with OCT images for marker identification in DR^7,27^.

In the case of OCTA, classic machine learning algorithms such as random forests or image feature analysis have been used before^28–31^. A large number of different types of neural networks have been trained on OCTA images for the diagnosis of DR. These approaches range from evaluating textural features, and transfer learning to ensembled approaches and the segmentation of vascular features^32–36^. Ryu et al. included a U-net in their approach for the diagnosis of DR^37^. There is no published work, to the best of our knowledge, that uses a U-Net to segment MAs from OCTA en face projections. This extends to ensembling of U-Nets as well as to the segmentation of MAs on two capillary layers at the same time.

Neural networks are commonly associated with the “black box” problem. If a network is trained on images with only a specific referral class as network output then the output is often not immediately traceable or explainable to the outside observer^38^. This is especially the case when a network generates a diagnosis or a referral without indicating which specific markers led to it. For this reason we propose to detect commonly recognized markers that indicate disease progression. This allows to leverage the adaptability of a neural network while generating segmentation results on markers that clinical staff can interpret and judge themselves^39,40^.

Because of OCTA being non-invasive and its ability to resolve different layers of the retina separately and the proven capabilities of the U-net architecture for biomedical segmentation tasks, we decided to adapt nnU-Net to the task of segmenting MAs from OCTA en face projections of the SCP and DCP. Creating an annotated data set with accurate labels is a very time-consuming process. We decided to approach that challenge by annotation MAs using bounding boxes that are converted to a binary label before training of the networks.

It is the objective of this work to detect MAs as an early marker for DR from OCTA scans, which can be acquired quickly and non-invasively, using an adapted U-net architecture. The paper is structured as follows: first we describe the creation of the expert labeled database and the adaptation of nnU-Net. Then we describe the evaluation, followed by a result and discussion section and a conclusion.

## Method

This section consists of two main parts. First, the MA labeling process and creation of the expert labeled database are described, followed by an explanation of the neural network.

This study was approved by the institutional review board (IRB) at Tufts Medical Center and conformed to the tenets of the Declaration of Helsinki and the Health Insurance Portability and Accountability Act of 1996. Informed consent obtained from patients at the New England Eye Center was considered exempt by the IRB because of the study’s retrospective design.

### Expert labeled database

Training of the network requires a training data set with accurate annotations. There is currently, to the best of our knowledge, no data set of OCTA scans of patients with NPDR/PDR and annotated MAs publicly available. We created a suitable data set ourselves, which was labeled by two expert graders from the New England Eye Center at Tufts Medical Center in Boston.

119 eyes of 70 patients diagnosed with early, intermediate, or severe NPDR or PDR were included in this study. Data were collected from a Zeiss Plex Elite 9000 SS-OCT device with dual-speed 100 kHz and 200 kHz A-scan rate, a lateral resolution of $\le 20$ micrometer, and an axial resolution of 6.3 micrometer. All OCTA images had a signal strength of 6 or grater indicated by the system’s software and were qualitatively screened for overall quality and excessive artifacts. The field size of all scans is $6\times 6$ mm.

**Table 1:** Number of eyes by disease stage in the test and training data sets. The separation into test and training data sets was randomized.

Diagnosis	Training eyes	Test eyes
Mild NPDR	13	4
Moderate NPDR	18	2
Severe NPDR	10	3
PDR	55	14
Total	96	23

The data was split into 96 eyes (from 52 patients) for training and 23 eyes (from 18 patients) for testing. The system software was used to segment the SCP and DCP of the OCTA scans and to generate en face projections. Table 1 shows the number of patients and diagnosis for the test and training data split.

**Fig. 1:**
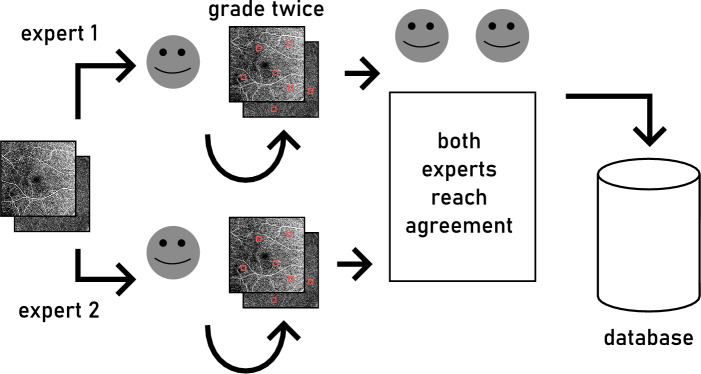
Labeling workflow of the two expert graders during the first stage based on Bertram et al.^41^. Each expert reviews the available en face projections (superficial and deep capillary plexuses) of every eye twice independently of each other. Afterwards they review the data together and need to come to an agreement on each MA. The resulting expert labeled database is used for initial training. The resulting false positives and false negatives were used by the graders during the second stage of the labeled database creation.

Our two stage approach for creating an *expert labeled database* of MAs from the SCP and DCP layers is similar to the one by Bertram et al.^41^. For the *first stage*, the two expert graders labeled MAs in the en face projections as is shown in Fig. 1. To create the expert labeled database, the graders used the open source web-based labeling tool EXACT to label MAs in both layers^42^. Each MA was annotated by creating a bounding box containing it on the en face projections. Even though MAs were annotated separately in the SCP and DCP, the presented method uses 2D images and 2D convolutions and not volumetric data. Each eye uses 2D fundus images, with the SCP and the DCP being in separate channels. First, the experts labeled MAs in the en face projections independently of each other by reviewing each en face projection image twice. MAs were identified by the experts by examining the available OCTA en face images themselves.

In the next step, the experts had to come to an agreement on each MA label which is necessary for it to become part of the expert labeled database. Only the bounding boxes on which both experts agreed remained in the database. In order to illustrate the challenge of finding and annotating all MAs, we computed the Pearson correlation coefficient for the numbers of MAs per eye labeled by each grader before they had to come to an agreement. How many MAs are labeled by each grader on a given eye can serve as a surrogate for reader agreement. The Pearson correlation coefficient for number of MAs labeled per eye is $\approx 0.21$ with a p-value of $\approx 0.045$. A correlation coefficient of 1.0 would indicate perfect agreement, while 0.0 indicates no correlation at all. This helps to illustrate the challenge for readers with respect to finding and annotating MAs.

**Fig. 2:**
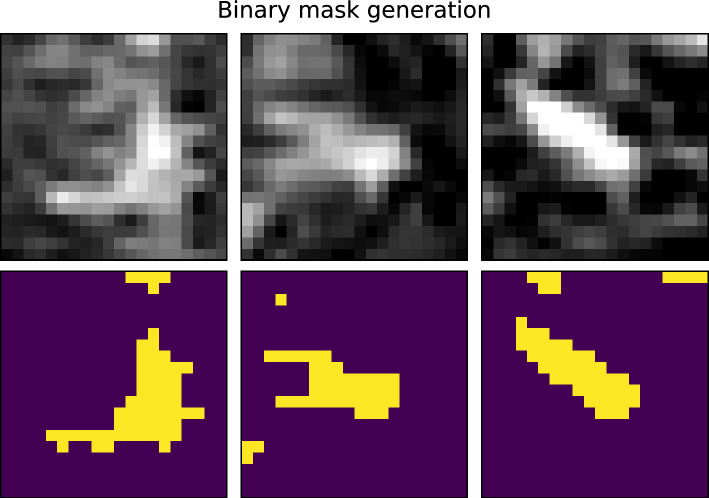
Example of how MA areas are converted to binary labels. The top row shows three different examples of MAs within bounding boxes. The values range from 0 (black) to 255 (white). The bottom row shows the corresponding binary masks for MAs after applying a binary threshold of 150. The bright areas indicate part of a MA, the dark areas indicate background.

The contents of the bounding boxes in the en face images were converted to training targets for the network via a thresholding step with examples shown in Fig. 2. A threshold of 150 was applied to the areas enclosed by bounding boxes to generate binary labels for the MAs. This threshold was chosen for the value range of 0 to 255 for the en face images. In order to find this threshold, a small subset of randomly chosen MAs was used to find a threshold that preserved the area of the MAs after thresholding. It is possible that small groups of unconnected pixels, not directly belonging to the MA remain, as seen in Fig. 2. This can be compensated later on by suppressing connected components below a given size on the network’s output (details further down below).

**Fig. 3:**
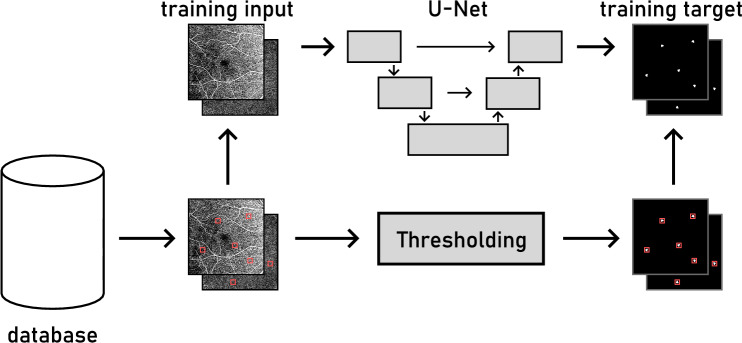
Ground truth target generation from the expert labeled database. The database contains the fundus projections and MA bounding boxes crated by the expert labelers (red rectangles). SCP and DCP are used as two-channel network input. The areas within the bounding boxes have a threshold applied to them and serve as binary targets for the network with one channel representing the SCP and other the DCP.

The OCTA en face projections were used as training input for the network, while the bounding box annotations were converted to binary ground truth images with per-pixel annotations of MAs for the network output. The process is shown in Fig. 3. The en face images served as input to the network, while the binary masks generated from the en face images and the bounding boxes were used as training targets. Both SCP and DCP en face projections were used as input for the network at the same time. The input used channel one for the SCP and channel two for the DCP.

The database was used for the first stage of training of the networks and to decide on the training parameters. After training this initial network on the training data set via fivefold cross-validation the *second stage* of the database creation could proceed. The resulting false positives (FPs) and false negatives (FNs) were then reviewed by the expert graders again. Because of the small size of potential MAs and their potentially large numbers, it is a challenge for the graders to find all MAs. Reviewing MAs, which were flagged by the neural network as false positives, can help to identify MAs that have been overlooked by the experts before. Even though the number of MAs in a given eye can be substantial, the overall fraction of all pixels in all images that belong to a MA is relatively small. This means that less than 1 % of all pixels were labeled as belonging to a MA. The database resulting from this two stage process was used for training of the networks and their results in the results and discussion section below.

**Fig. 4:**
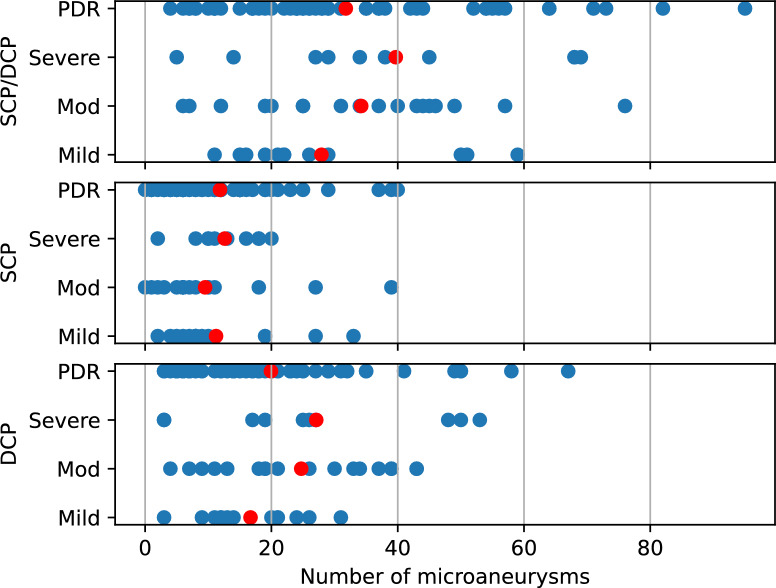
Number of labeled MAs per eye categorized by mild, moderate, and severe NPDR and PDR. The blue markers indicate the number of MAs on an eye of the given DR severity. The red markers indicate the mean number of MAs each.

In order to assert the quality of the labeled data set, we show the number of labeled MAs per eye in Fig. 4. The diagram indicated the number of MAs labeled per eye with the given disease severity. I.e. the blue marker near 60 indicated that an eye with the diagnosis of mild NPDR contains 59 labeled MAs. The red markers indicate increasing numbers of MAs coinciding with disease progression. There is a drop from severe NPDR to PDR however, which is likely related to laser treatment in patients. Furthermore, the graders annotated more MAs in the DCP than in the SCP. This is consistent with previous studies, which state that MAs occur more often in the DCP^4,43^.

### U-Net

We decided to use a U-net, first published by Ronneberger at al., to segment MAs due to its proven effectiveness for medical segmentation tasks^9^. It consists of a convolutional down-sampling branch which downsizes the image data while computing features using the filters defined during training. It is completed by an up-sampling branch in order to provide per-pixel labels that match the size of the input. The number of down-sampling steps depends on the size of the input images and structures to be segmented while the intermediate feature maps from the down-sampling branch are also passed on to the up-sampling branch. This preserves spatial information that could otherwise be lost during subsequent down-sampling operations. The combination of these elements makes the U-net architecture a proven network design and candidate for the segmentation of MAs^9,23–25^.

We use nnU-Net as a starting point for our U-net adaptation for MA segmentation. nnU-Net is a generalized toolbox that specializes in providing support for solving segmentation problems in biomedical imaging. It provides a U-net adapted automatically to the dimensions of the images to be trained on. It additionally provides a sane set of default settings and heuristic rules based on properties of the data set. nnU-Net differentiates between three different sets of parameters. The first set is comprised of parameters that remain the same across all potential segmentation tasks, e.g. the U-net architecture, but also the optimizer and its learning rate, number of epochs, the loss function and augmentations. The second set is rule-based and based on the properties of the training data, e.g. intensity distribution, spacing of pixels, and modality (e.g. computed tomography). The third set of parameters is empirical. This means that nnU-Net can make certain choices based on post-processing. The advantage of nnU-Net is that it provides a deep learning pipeline that should lead to usable results without additional changes. Its defaults however, leave room for changes and additional tuning to improve the results delivered by nnU-Net. Additionally, nnU-Net supports ensembling of trained networks. I.e., if enough data are available for a train/test split, the five networks trained on each of the cross-validation folds can be used as an ensemble on the test data. For this, the output of the five nets are averaged. This can lead to an improvement in segmentation performance at the expense of increased training time. nnU-Net’s architecture uses skip-connections to avoid over-fitting, a combination of dice and cross-entropy loss, leaky ReLUs as activation function, deep supervision, and it uses stochastic gradient descent with Nesterov momentum for training^9,44^.

Due to the imbalance of the expert labeled database (less than 1% of pixel belong to a MA), we decided to investigate focal loss and dice loss and compared them with the default nnU-Net configuration^45^. We also added a comparison with TransUNet and Swin-Unet, which are two state-of-the-art U-net implementations. TransUNet adds transformers and pre-trained weights to the U-net architecture^46^, while Swin-Unet implements a transformer-based U-shaped encoder-decoder architecture with skip-connections for local-global semantic feature learning^47^. Additionally, we suppressed connected components with a width or height of less than 11 pixels to reduce the number of false positives. All configurations were trained with a learning rate of 0.1.

## Results and discussion

We provide both per-pixel and per-MA metrics as part of the evaluation. The metrics per pixel show how many pixels are classified correctly as belonging to a MA or not while the per-MA metrics indicate whether a MA was picked up by a net or not or whether the net detected a false positive MA. Even though the per-pixel metrics help to understand the overall results, we consider the per-MA metrics to be the more clinically relevant metric. Furthermore, we have added comparisons with TransUNet and Swin-Unet^3,46^. Both network architectures serve as a point of reference for the changes we have made to nnU-Net.

Overall, we compare three U-Net configurations, TransUNet, and Swin-Unet:

- the original nnU-Net configuration
- a new configuration using dice loss,
- a new configuration using focal loss, and
- TransUNet, which is a state-of-the-art implementation of the U-net architecture adding transformers and pre-trained weights.
- Swin-Unet, which is a state-of-the-art implementation of the U-net architecture adding a transformer-based U-shaped encoder-decoder architecture with skip-connections for local-global semantic feature learning.

Since FA is the gold standard for the diagnosis of DR and MAs, it seems self-evident to use FA images for the evaluation of any automated detection algorithm. The challenge to this approach lies in the dynamic nature of MAs themselves. The number of MAs can vary from visit to visit^3^. Both OCTA scans and FA images would need to be be acquired during the same visit. Due to the difficulty of of obtaining OCTA scans and FA images from the same visit, we rely on a comparison to state-of-the-art networks instead.

We list precision/recall and associated metrics (number of true positives, false negatives, false positives, F1-score) for each configuration. For per-pixel results we provide area-under-curve (AUC), and precision/recall metrics.

**Fig. 5:**
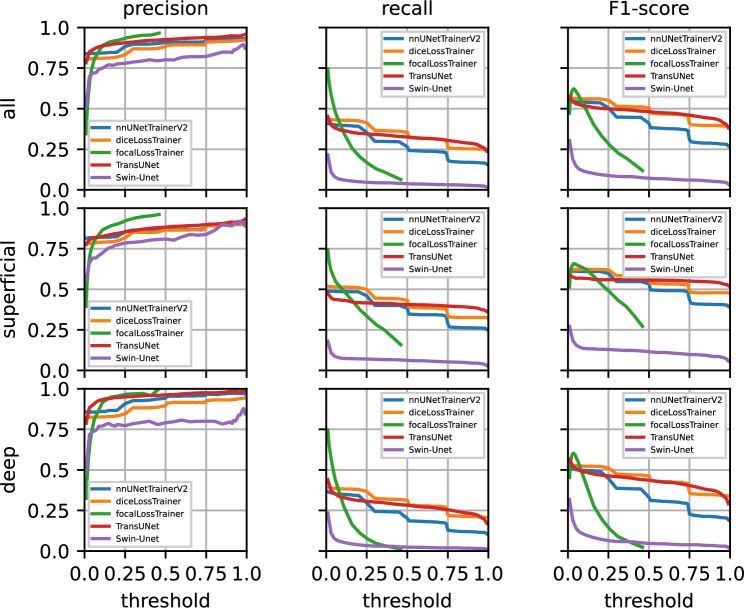
From left to right: precision, recall, and F1-score curves over five training folds on the training data. The top row shows the results across both layers, with results for superficial and deep capillary plexuses shown separated below. Each neural network’s results are shown in a different color.

**Fig. 6:**
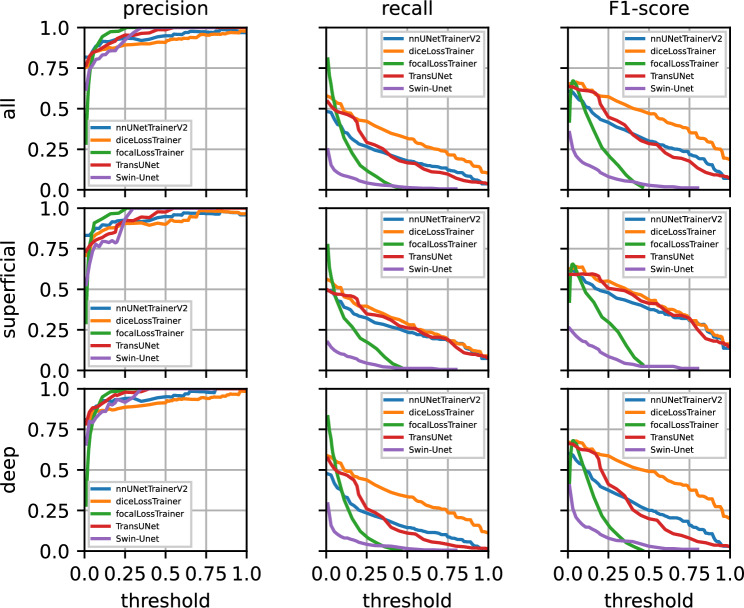
From left to right: precision, recall, and F1-score curves using ensembling on the test data. The top row shows the results across both layers, with results for superficial and deep capillary plexuses shown separated below. Each neural network’s results are shown in a different color.

Figure 5 shows precision/recall curves using fivefold cross-validation on the training data over the decision thresholds. Table 2 shows results for the same data at different decision thresholds. Figure 6 and Table 3 show results on the test data using an ensemble of the five U-nets, five TransUNets, and five Swin-Unets trained on each of the fivefolds of the training data.

**Table 2:** Results for fivefold cross-validation on the training data.

		Thresh	Per microaneurysm						Per pixel			
			TPs	FNs	FPs	Prec	Rec	F1	AUC	Prec	Rec	F1
SCP	nnU-Net	0.05	534	560	118	0.82	0.49	0.61	0.73	0.44	0.47	**0.45**
		0.25	503	591	85	0.86	0.46	0.60	0.70	0.50	0.41	**0.45**
		0.45	438	656	68	0.87	0.40	0.55	0.68	0.55	0.36	0.44
		0.99	276	818	29	0.90	0.25	0.39	0.60	0.65	0.21	0.32
	Dice	0.05	564	530	154	0.79	0.52	0.62	0.74	0.42	0.48	**0.45**
		0.25	546	548	122	0.82	0.50	0.62	0.73	0.45	0.45	**0.45**
		0.45	483	611	84	0.85	0.44	0.58	0.70	0.51	0.40	**0.45**
		0.99	355	739	39	0.90	0.32	0.48	0.63	0.64	0.27	0.38
	Focal	0.05	**621**	**473**	185	0.77	**0.57**	**0.65**	**0.75**	0.34	**0.50**	0.41
		0.25	362	732	31	0.92	0.33	0.49	0.61	0.62	0.21	0.31
		0.45	179	915	7	**0.96**	0.16	0.28	0.54	**0.71**	0.08	0.14
	TransUNet	0.5	444	650	59	0.88	0.41	0.56	0.61	0.39	0.23	0.29
		0.25	455	639	78	0.85	0.42	0.56	0.62	0.37	0.24	0.29
		0.45	446	648	61	0.88	0.41	0.56	0.62	0.39	0.23	0.29
		0.99	397	697	29	0.93	0.36	0.52	0.58	0.46	0.17	0.24
	Swin-Unet	0.5	68	1026	16	0.81	0.06	0.12	0.51	0.17	0.03	0.05
		0.25	77	1017	21	0.79	0.07	0.13	0.52	0.16	0.04	0.06
		0.45	71	1023	17	0.81	0.06	0.12	0.52	0.17	0.03	0.05
		0.99	33	1061	**4**	0.89	0.03	0.06	0.50	0.20	0.01	0.02
DCP	nnU-Net	0.05	719	1309	121	0.86	0.35	0.50	0.58	0.20	0.15	0.17
		0.25	602	1426	61	0.91	0.30	0.45	0.56	0.24	0.11	0.15
		0.45	487	1541	33	0.94	0.24	0.38	0.54	0.28	0.09	0.13
		0.999	181	1847	5	0.97	0.09	0.16	0.51	0.42	0.02	0.03
	Dice	0.05	783	1245	166	0.83	0.39	0.53	0.60	0.18	0.20	**0.19**
		0.25	745	1283	137	0.84	0.37	0.51	0.59	0.20	0.18	**0.19**
		0.45	646	1382	83	0.89	0.32	0.47	0.58	0.22	0.15	0.18
		0.99	418	1610	25	0.94	0.21	0.34	0.54	0.28	0.09	0.13
	Focal	0.05	**948**	**1080**	293	0.76	**0.47**	**0.58**	**0.63**	0.15	**0.26**	**0.19**
		0.25	155	1873	5	0.97	0.08	0.14	0.51	0.43	0.02	0.05
		0.45	28	2000	**0**	**1.00**	0.01	0.03	0.50	**0.65**	0.00	0.01
	TransUNet	0.5	573	1455	22	0.96	0.28	0.44	0.52	0.18	0.04	0.07
		0.25	621	1407	33	0.95	0.31	0.46	0.53	0.17	0.05	0.08
		0.45	580	1448	25	0.96	0.29	0.44	0.52	0.18	0.05	0.07
		0.99	347	1681	4	0.99	0.17	0.29	0.51	0.23	0.02	0.03
	Swin-Unet	0.5	49	1979	13	0.79	0.02	0.05	0.50	0.08	0.01	0.01
		0.25	68	1960	20	0.77	0.03	0.06	0.50	0.08	0.01	0.02
		0.45	53	1975	15	0.78	0.03	0.05	0.50	0.08	0.01	0.01
		0.99	22	2006	4	0.85	0.01	0.02	0.50	0.12	0.00	0.01

Results for the three tested configurations (nnU-Net, dice, and focal loss) and TransUNet and Swin-Unet are shown both per MA and per pixel. Numbers shown are the combined results across all fivefolds. The best result in each column is marked in bold.

**Table 3:** Results for ensembled classification on the test data.

		Thresh	Per microaneurysm						Per pixel			
			TPs	FNs	FPs	Prec	Rec	F1	AUC	Prec	Recall	F1
SCP	nnU-Net	0.05	146	167	27	0.84	0.47	0.60	0.73	0.45	**0.47**	**0.46**
		0.25	101	212	8	0.93	0.32	0.48	0.66	0.66	0.32	0.43
		0.45	80	233	5	0.94	0.26	0.40	0.63	0.73	0.25	0.38
		0.99	23	290	1	0.96	0.07	0.14	0.53	0.84	0.05	0.10
	Dice	0.05	**169**	**144**	52	0.76	**0.54**	**0.63**	**0.77**	0.36	0.55	0.43
		0.25	124	189	13	0.91	0.40	0.55	0.68	0.54	0.37	0.44
		0.45	98	215	10	0.91	0.31	0.47	0.64	0.62	0.29	0.39
		0.99	28	285	1	0.97	0.09	0.16	0.54	0.78	0.09	0.16
	Focal	0.05	151	162	25	0.86	0.48	0.62	0.72	0.40	0.43	0.42
		0.25	54	259	**0**	**1.00**	0.17	0.29	0.56	0.83	0.13	0.22
		0.45	7	306	**0**	**1.00**	0.02	0.04	0.51	**0.98**	0.01	0.02
	TransUNet	0.05	148	165	40	0.79	0.47	0.59	0.68	0.30	0.36	0.33
		0.25	109	204	9	0.92	0.35	0.51	0.62	0.50	0.24	0.32
		0.45	86	227	2	0.98	0.27	0.43	0.58	0.60	0.16	0.25
		0.99	25	288	**0**	**1.00**	0.08	0.15	0.52	0.84	0.03	0.06
	Swin-Unet	0.05	39	274	14	0.74	0.12	0.21	0.54	0.11	0.07	0.09
		0.25	14	299	1	0.93	0.04	0.09	0.51	0.23	0.02	0.04
		0.45	4	309	0	1.00	0.01	0.03	0.51	0.42	0.01	0.02
		0.99	1	312	0	1.00	0.00	0.01	0.50	0.93	0.00	0.00
DCP	nnU-Net	0.05	224	310	41	0.85	0.42	0.56	0.61	0.19	0.22	0.20
		0.25	125	409	8	0.94	0.23	0.37	0.55	0.28	0.10	0.15
		0.45	86	448	5	0.95	0.16	0.28	0.53	0.32	0.06	0.10
		0.99	8	526	**0**	**1.00**	0.01	0.03	0.50	0.43	0.00	0.01
	Dice	0.05	**304**	**230**	73	0.81	**0.57**	**0.67**	**0.64**	0.15	0.29	0.20
		0.25	234	300	30	0.89	0.44	0.59	0.59	0.21	0.19	0.20
		0.45	188	346	19	0.91	0.35	0.51	0.57	0.23	0.14	0.17
		0.99	61	473	1	0.98	0.11	0.20	0.52	0.26	0.03	0.06
	Focal	0.05	**304**	**230**	78	0.80	**0.57**	0.66	**0.64**	0.16	0.28	0.20
		0.25	47	487	**0**	**1.00**	0.09	0.16	0.51	0.34	0.02	0.05
		0.45	2	532	**0**	**1.00**	0.00	0.01	0.50	**0.49**	0.00	0.00
	TransUNet	0.05	271	263	36	0.88	0.51	0.64	**0.57**	0.13	0.14	0.14
		0.25	139	395	3	0.98	0.26	0.41	0.52	0.23	0.04	0.07
		0.45	66	468	**0**	**1.00**	0.12	0.22	0.51	0.30	0.02	0.03
		0.99	8	526	**0**	**1.00**	0.01	0.03	0.50	0.48	0.00	0.00
	Swin-Unet	0.05	64	470	17	0.79	0.12	0.21	0.52	0.11	0.05	0.07
		0.25	22	512	2	0.92	0.04	0.08	0.51	0.21	0.01	0.03
		0.45	12	522	**0**	1.00	0.02	0.04	0.50	0.24	0.01	0.01
		0.99	0	534	**0**	N/A	0.00	N/A	0.50	0.00	0.00	0.00

Results for the three tested configurations (nnU-Net, dice, and focal loss) and TransUNet and Swin-Unet are shown both per MA and per pixel. The best result in each column is marked in bold.

First, we consider the fivefold cross-validation results on the training data. For each nnU-Net configuration, including the default nnU-Net and our adaptations with dice loss and focal loss, a single network was trained on each fold. TransUNet and Swin-Unet were also trained once for every one of the fivefolds. Figure 5 and Table 2 show these results.

First of all, it is apparent that the curves in Fig. 5 for the default nnU-Net and the dice loss version behave similarly due to nnU-Net’s loss being a combination of dice loss and cross-entropy loss. The precision is slightly lower for dice loss, but the recall is better for dice loss when compared to nnU-Net. This does not come at a surprise considering the class imbalance in the data set and the fact that cross-entropy loss does not perform well on imbalanced data sets without compensating features such as sample weights. The precision of TransUNet is also higher when compared to the dice loss configuration, but the recall is worse for lower thresholds and slightly better for higher thresholds. This extends to the F1-scores. Focal loss, on the other hand, achieves the highest precision. It also displays the the highest recall at low thresholds but this coincides with very low precision. Precision across all networks is noticeably better in the DCP, when compared to the SCP. The opposite applies to the recall across all networks. It is generally higher in the SCP, when compared to the DCP. Swin-Unet however, consistently shows worse precision and recall when compared to the other networks.

Next, we evaluate the results of the ensembled networks on the test data in Fig. 6 and Table 3. Several of the previous observations from the results on the fivefold cross-validation data still hold true. Precision for the dice loss configuration is slightly worse than for the nnU-Net configuration. The precision of TransUNet is higher than both dice loss and default nnU-Net configurations. Again, the focal loss configuration performs best in the lower decision threshold ranges, but the F1-score is in the same range as the other configurations. The precision across all networks is slightly better in the SCP when compared to the DCP. Recall decreases in the DCP when compared to the SCP, except for the dice loss configuration. Interestingly, it appears that the dice loss network benefits from the ensembling of networks, which is a notable exception to the other networks. When considering the F1-scores on the SCP, dice loss and TransUNet show very similar performances overall, with the dice loss performing slightly better. This changes in the case of the DCP however, with the dice loss’ improved recall also improving its F1-score. Swin-Unet shows improved precision when used with ensembling on the test data, its recall, however, does not notably improve.

When comparing the results for the cross-validation evaluation on the training data in Table 2 with the ensembled results on the test data in Table 3, it becomes clear that precision improves across all tested configurations for the ensembled networks. Recall however, increases for lower thresholds while it decreases for higher thresholds with the exception for dice loss in the DCP. Generally, a decrease in recall is unfortunate for use cases such as screening, where high recall (e.g., finding every possible case of the condition) is preferred over precision, to ensure as few cases as possible are missed. Note that in screening scenarios, it’s often more important to identify all possible cases (high recall) rather than being overly concerned about false positives (high precision). This is because missing a true case (a false negative) can have more severe consequences than incorrectly identifying a case that isn’t there (a false positive), which can usually be ruled out with further testing.

**Fig. 7:**
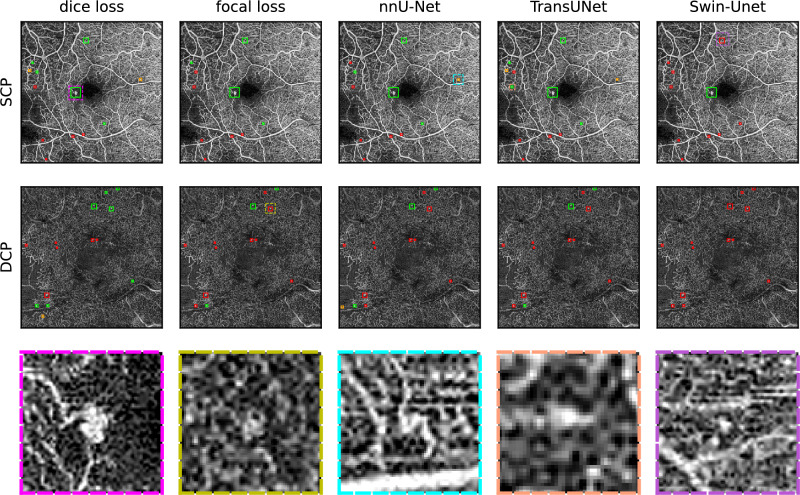
Patient 1: MA segmentation results on superficial (top row) and deep capillary plexuses (middle row) of an eye with PDR using dice loss, focal loss, default nnU-Net, TransUNet, and Swin-Unet ensembles. Green boxes indicate MAs that were correctly identified by the U-net ensemble. Red boxes indicate false negatives and orange boxes indicated false positives. The areas with dashed outlines are shown enlarged in the bottom row.

**Fig. 8:**
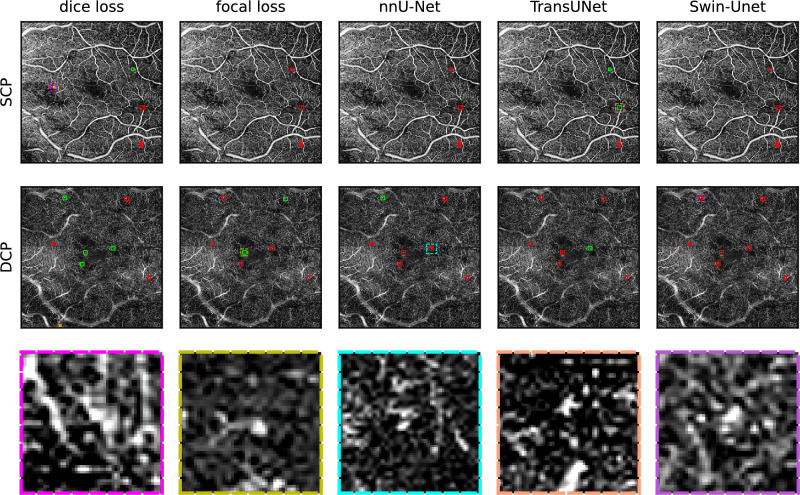
Patient 2: MA segmentation results on superficial (top row) and deep capillary plexuses (middle row) of an eye with PDR using dice loss, focal loss, default nnU-Net, TransUNet, and Swin-Unet ensembles. Green boxes indicate MAs that were correctly identified by the U-net ensemble. Red boxes indicate false negatives and orange boxes indicated false positives. The areas with dashed outlines are shown enlarged in the bottom row.

For both sets of results, the fivefold cross-validation on the training data and ensembling on the test data, it is apparent that precision is higher in the DCP. We mainly attribute this to the difference in vascular morphology between the two layers. OCTA scans of the SCP show clear and continuous vessel shapes against a black background, while the DCP shows a greater similarity to a regular distribution and small complex interconnections^48^. This can be observed in Figs. 7 and 8. Also, for both sets of results, recall for the DCP decreases when compared to the SCP. Even though fewer FPs in the DCP benefit precision, we theorize that the larger number of annotated MAs in the DCP lead to slightly fewer of them being found and thus inhibiting recall. On the training data set, 1094 MAs were annotated in the DCP, while 2028 MAs were annotated on the DCP, almost twice as many. On the test data set, 313 MAs were annotated in the SCP, while 534 MAs were annotated in the DCP. This is congruent with the clinical observations in DR, where the majority of MAs tend to occur in the DCP, not the SCP^4,43^. A somewhat reduced recall in the DCP can be compensated for by the larger number of MAs in that layer, as long as the recall does not sink too closely towards 0 (see Tables 2 and 3. For instance, in case of the dice loss on the test data in Table 3, the recall is still 0.35 at a decision threshold of 0.45 at a precision of 0.91 with 188 MAs found out of 534.

The ensembling step works by running the prediction of the five networks, each trained on a different fold of the training data, on the test data. The five predictions for each eye are then averaged. Dice loss in the DCP benefits from this step disproportionately when compared to the other networks and their losses and compared to the SCP. In the case of TransUNet for instance, it is possible that each of the five instances find different subsets of MAs in the DCP, but those fall below the size and decision threshold when ensembled. Dice loss on the DCP by comparison, performs better in this instance due to a combination of the tendency to favor contained areas with clearly delineated outlines and its resilience toward class imbalance. This is illustrated in supplementary Fig. F1, which shows the network output for patients 1 and 2 shown in Figs. 7 and 8 respectively.

Overall, nnU-Net’s default configuration, the dice loss configuration, and TransUNet behave very similarly due to nnU-Net’s and TransUNet’s loss being a combination of dice and cross-entropy loss. This can be seen in Figs. 5 and 6. The fact that the dice loss configuration achieves a better recall than nnU-Net does not come at a surprise considering the class imbalance in the data set and the fact that cross-entropy loss does not perform well on imbalanced data sets without features that compensate for it, such as sample weights. The changes to TransUNet over nnU-Net however, are able to partially compensate for this.

Figure 7 shows en face projections of the SCP and DCP from a patient’s eye with PDR and macular edema. A true positive from the SCP using dice loss is enlarged in the lower left. This is a large MA that was found by all five neural networks. A false negative MA from the DCP is shown in the bottom center left. Even though this is an annotated MA, it has only been found by U-net ensemble using the dice loss configuration. The lower center right shows a false positive that has been found by the default nnU-Net configuration in the SCP. The enlarged area shows a potential vascular anomaly that could be an MA, that was not labeled. The lower right shows an MA from the SCP that was only found by the TransUNet ensemble. Figure 8 shows en face projections of the SCP and DCP from another patient’s eye with PDR and macular edema. A false positive from the SCP using dice loss is enlarged in the lower left. A true positive MA from the DCP is shown in the bottom center left. This MA has been found by the dice and focal loss nnU-Net configurations but not by the default nnU-net. The lower center right shows a false negative that has not been found by the default nnU-Net configuration in the DCP. This MA could be found using the dice loss configuration. The lower right shows another MA that was only found by the TransUNet ensemble.

## Conclusion and outlook

In this paper we present two things. First, we created a data set of MAs on the SCP and DCP OCTA projections from patients with DR for the training and evaluation of U-nets by two expert graders in two rounds of labeling. Secondly, we present different U-net configurations designed to detect MAs in en face projections of the SCP and DCP from OCTA scans of patients with DR and compare them with TransUNet and Swin-Unet. Our results demonstrate that it is possible to detect MAs with high accuracy/specificity albeit at the cost of recall/sensitivity. Even though higher recall is preferable in a clinical screening scenario, it never reaches zero in case of the presented dice loss configuration. The performance of the networks is generally comparable for application on the SCP and DCP, with the former benefiting from higher recall and the latter from slightly higher precision. The dice loss configuration is also the only network that benefited from ensembling in the DCP due to its resilience toward class imbalance and its ability to highlight clearly delineated areas. Overall, we demonstrate the viability of the U-net architecture for the segmentation of MAs in both the SCP and DCP in patients with DR. Using markers that are recognizable avoids the “black box” problem commonly associated with deep learning and allows clinicians to evaluate and trace the diagnosis made by the system.

Future work will include additional recognized markers, such as measurement/segmentation of non-perfused areas and foveal avascular zone enlargement^3,5^, a larger data set, and will aim for making referable predictions based on specific disease markers.

## Supplementary Information

Supplementary Figure 1.

## Data Availability

Data underlying the results presented in this paper are not publicly available at this time but may be obtained from the corresponding author upon reasonable request.
