# Supplementary material for: Ensembling U-Nets for microaneurysm segmentation in optical coherence tomography angiography in patients with diabetic retinopathy
Source: Sci Rep. 2024 Sep 14;14:21520. doi: 10.1038/s41598-024-72375-2 (PMC11401926; doi:10.1038/s41598-024-72375-2)
Supplement: Supplementary file 1 — Supplementary Figure 1. [file 41598_2024_72375_MOESM1_ESM.pdf]

Supplementary Figure F1

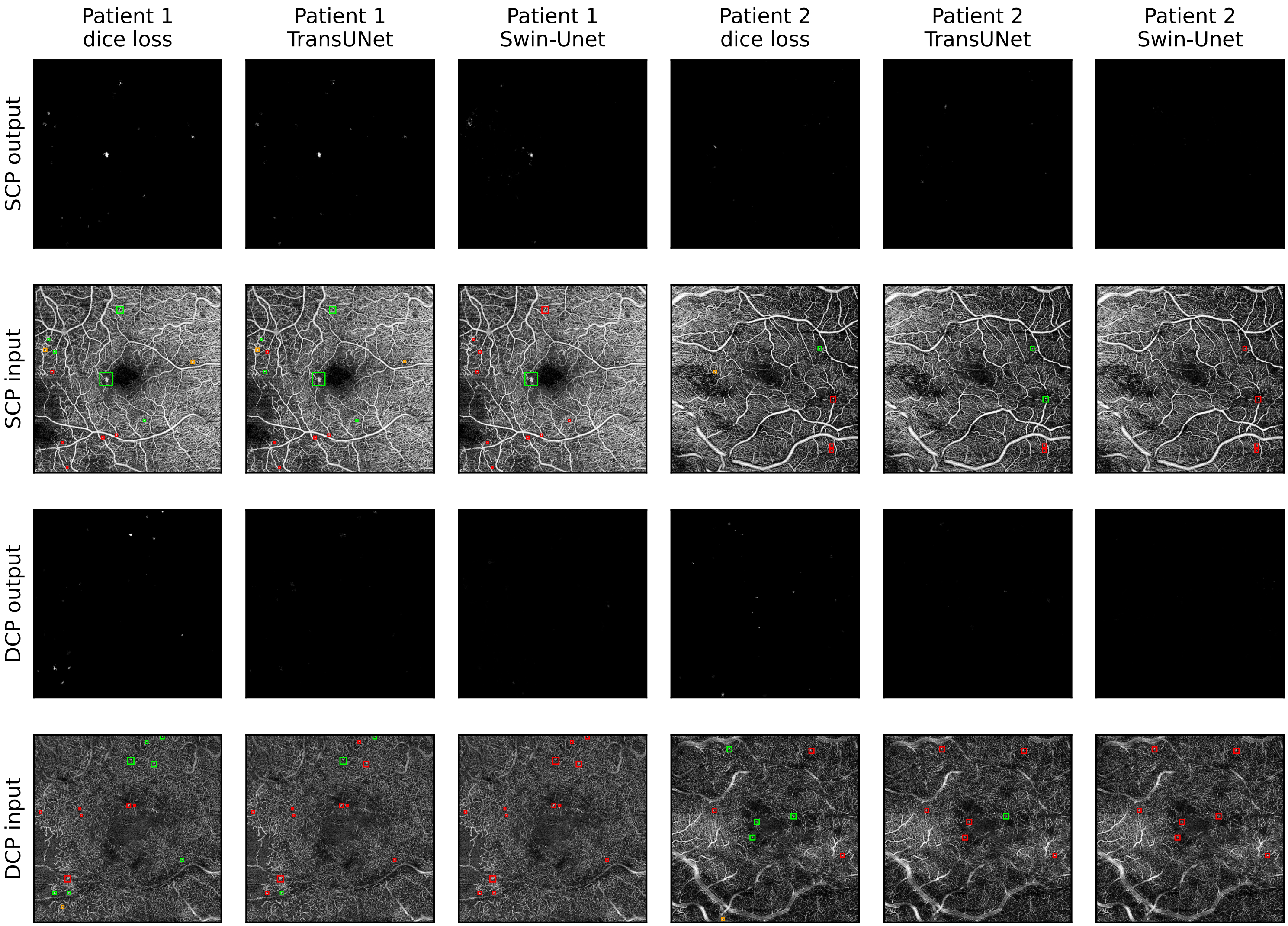

Comparison of network output: the three left columns show patient 1 (see Figure 7), the three right columns show patient 2 (see Figure 8). The rows show, from top to bottom, SCP output, SCP input, DCP output, and DCP input. The dice loss, TransUNet, and Swin-Unet results are shown for each patient. The input images show the detected true positives (green boxes), false negatives (red boxes) and false positives (orange boxes) for a decision threshold of 0.3 and a minimum size for an MA of 11 by 11 pixels. The better performance of ensembled dice loss in the DCP can be seen in the third row, compared to the ensembled TransUNet and Swin-Unet outputs.
